# Modeling the interactions of Alzheimer-related genes from the whole brain microarray data and diffusion tensor images of human brain

**DOI:** 10.1186/1471-2105-13-S7-S10

**Published:** 2012-05-08

**Authors:** Byungkyu Park, Wook Lee, Kyungsook Han

**Affiliations:** 1 Institute for Information and Electronics Research, Inha University, Incheon, South Korea; 2 School of Computer Science and Engineering, Inha University, Incheon, South Korea

## Abstract

**Background:**

In recent years the genome-wide microarray-based gene expression profiles and diffusion tensor images (DTI) in human brain have been made available with accompanying anatomic and histology data. The challenge is to integrate various types of data to investigate the interactions of genes that are associated with specific neurological disorder.

**Results:**

In this study, we analyzed the whole brain microarray data and the physical connectivity of the hippocampus with other brain regions to identify the genes related to Alzheimer's disease and their interactions with proteins. We generated a physical connectivity map of the left and right hippocampuses with 12 other brain regions and identified 33 Alzheimer-related genes that interact with many proteins. These genes are highly linked to the development of Alzheimer's disease.

**Conclusions:**

In Alzheimer's brain both brain regions and inter-regional communications through the white matter are often hampered. So far the connectivity of regions in Alzheimer's brain has been studied mostly at the functional level using functional MRI (fMRI). Analyzing the inter-regional fiber connectivity without tracking crossing-fiber regions often provides coarse and inaccurate results. A few deep brain fibers were analyzed but the inter-regional fiber connectivity was not analyzed in their studies. The inter-regional fiber connectivity analysis can provide comprehensive and measurable degradation of fiber tracts in AD patients' brains, but is not easy to perform. We tracked crossing-fiber regions and identified genes with high expression levels in the fiber pathways of the hippocampus. The interactions of the genes with other proteins can provide comprehensive and measurable degradation of fiber tracts in Alzheimer brains. To the best of our knowledge, this is the first attempt to integrate the whole brain microarray data with DTI data to identify specific genes and their interactions.

## Background

Alzheimer's disease (AD) is one of the most devastating diseases for people in advanced age between 40 to 60 years. It affects parts of the brain that control thought, memory, and sometimes language. The most affected part is the memory which is primarily controlled by the hippocampus. Thus, a comprehensive study of the connectivity of the hippocampus with other brain regions may reveal a new pattern specific to AD.

So far the connectivity of regions in Alzheimer's brain has been studied mostly at the functional level using functional MRI (fMRI) [1,2]. It is until recently that the importance of the physical connectivity of regions in Alzheimer's brain has become evident [3-6]. The cause of AD is now believed to be the excessive storage of Amyloid Beta (Aβ) plaque [7] into the white fiber tracts. So, the study of relationship between fiber tracts and Amyloid Beta is very important. For the past few years diffusion tensor imaging (DTI) has received much attention from brain studies since it can capture the fine fiber orientations of brain white matter. Water molecules diffuse in the direction of the fiber more rapidly due to the myelination property of the tracts, and DTI records the diffusion. DTI including information of diffusion can provide inter-regional fiber pathway.

Each of the DTI data and the microarray data for a human brain takes much time and effort to generate. Thus, most DTI data was generated independently from brain microarray data so far. Recently both DTI and gene expression data were generated from two human brains that have no known neuropsychiatric or neuropathological history [8]. Thus, it is timely to track inter-region physical connectivity pathways from the DTI data and find Alzheimer-related genes expressed in the pathways to identify the interactions of the genes. Analyzing genes expressed in specific fiber pathways will help identify the interactions of genes in neurons, which in turn will provide a valuable resource to research and treatment of neurological disorders such as Alzheimer's disease.

In this study, we tracked inter-region fiber tracts using a new method for detecting crossing fibers in voxels and streamlined the fibers by probabilistic tractography [9] to generate an inter-region physical connectivity map of the hippocampus. The primary focus of this study is to investigate the connectivity of the left and right hippocampus with other brain regions. The secondary focus of this study is to find the interactions of Alzheimer-associated genes that are expressed in the fiber pathways of hippocampuses. The rest of this paper presents our method of tracking the fiber pathways and finding genes highly expressed in the fiber pathways and major results of our study.

## Methods

### Dataset

Both the brain microarray data and the DTI data for a 24 year old male were obtained from the Allen Brain Atlas (ABA, http://human.brain-map.org/). In the microarray, there are 58,692 probes to detect for human brain genes. We selected 473 genes that are known to be relevant with the Alzheimer disease. The diffusion tensor image (DTI) for the same brain was obtained from ABA.

To identify protein-protein interactions (PPIs) that involve brain genes, we extracted 39,194 PPIs from the Human Protein Reference Database (HPRD). 473 Alzheimer's disease genes are associated with 96 human proteins in HPRD. 81 out of the 96 proteins have 1,506 interactions.

### Human brain fiber tractography

**Figure 1:**
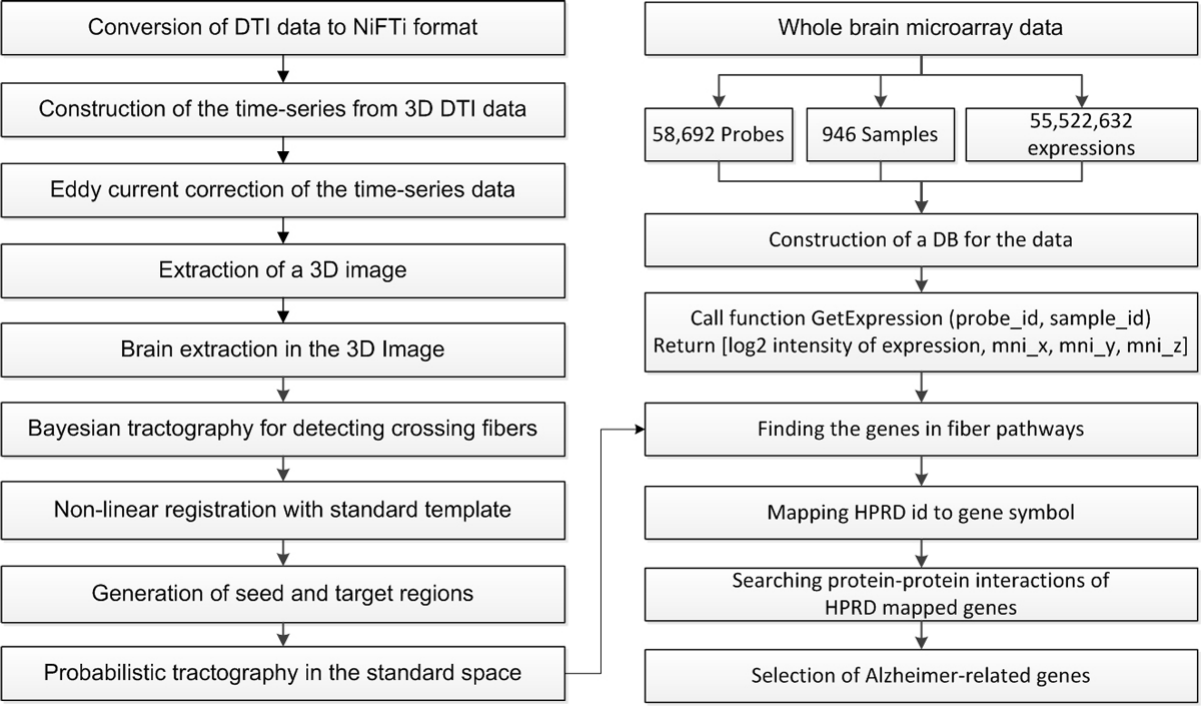
**Framework for the tractography**. The process of performing tractography from the DTI data and finding protein-protein interactions of the Alzheimer-related genes from the whole brain microarray data.

The left part of Figure 1 shows the process of performing tractography from the DTI data of human brain. First, we converted the DTI data from .hdr and .img formats to the FSL compatible NiFTi format (*.nii) using the LONI-Inspector (http://www.loni.ucla.edu/Software/LONI-Inspector) program. After conversion of data format, we used the FMRIB Software Library (FSL) for processing DTI. FSL provides a complete set of tools for the processing and analysis of DTI [10].

3D NiFTi files converted by FSL were merged into a single 4D NiFTi image. The 4D NiFTi image was corrected to remove distortions caused by eddy currents and simple head motion. From corrected 4D image, we extracted standalone 3D image with no diffusion. This 3D image was used as a reference after process. For the non-linear registration with the standard template, we discarded the skull from the reference 3D image using the Brain Extraction Tool (BET) [11]. After discarding the skull we obtained a pure brain without the skull.

Recently the technique of detecting crossing fiber regions that appear multiple principal diffusion direction at each voxel has been widely used for the analysis of DTI. We estimated direction of diffusion using the Bayesian Estimation of Diffusion Parameters Obtained using Sampling Techniques (BEDPOSTX) [9] to detect two crossing fibers at each voxel. BEDPOSTX made a multi fiber diffusion model using crossing fiber regions.

After detecting crossing fibers, we used the FMRIB's Non-linear Image Registration Tool (FNIRT) for registration. FNIRT generated a warp field and its corresponding inversed warp field for a tractography in the standard space. We selected the MNI152-T1 1mm template for the registration and the probabilistic tractography. The region of interest (ROI), which was used as the seed and target in fiber tracking, was defined in this standard template.

**Figure 2:**
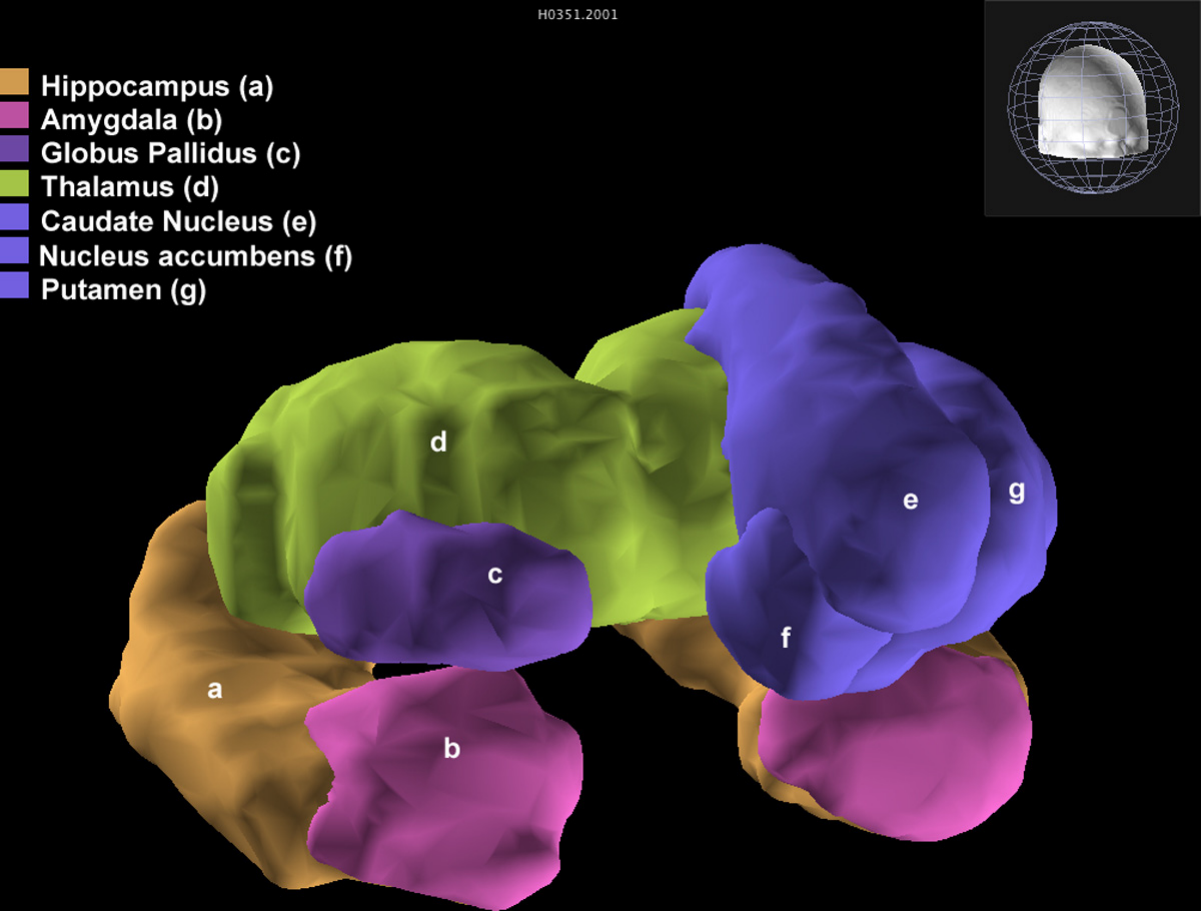
**Seed and target regions**. The brain-explorer of ABA can represents separated ROIs in the human brain. The left and right hippocampuses (orange) used as the seed regions and the target regions (pink, blue, purple and green).

The hippocampus is known to be closely related to learning and memory. The early symptom of Alzheimer's disease is memory impairment, so we chose the hippocampus for the seed region of probabilistic tractography. To identify seed and target ROIs, we used the FMRIB's Integrated Registration and Segmentation Tool (FIRST) [12] and extracted 15 ROIs including the hippocampus (left and right hippocampus, left and right nucleus accumbens, left and right amygdala, left and right caudate nucleus, left and right globus pallidus, left and right putamen, left and right thalamus, and brainstem) from the standard template (Figure 2). Tractography was then performed in the standard space from every voxel of the seed ROI for other all target ROIs except the brainstem. We traced the pathway by hemisphere (left to left and right to right). Thus we obtained 2 (left and right) × 1 (seed) × 6 (target) = 12 pathways.

### Finding samples in tractography

Each of the 946 samples of the human brain microarray data contains 58,692 probes. To identify the genes expressed in fiber pathways, we examined the positions of the voxels in the 946 samples, and selected the samples that are placed in the pathways. The selected samples contain 473 genes that are related to Alzheimer's disease, and 81 genes out of the 473 genes have protein-protein interaction data available at HPRD.

The human brain microarray data has the expression values of the genes as the log_2 _intensity. For each probe we computed the z-score by the following equation.

(1) $$z-score=\frac{x-\mu }{\sigma }$$

where *x *is the log_2 _intensity, *μ *is the mean, and *σ *is the standard deviation.

Since multiple samples can be placed in a pathway, a gene can have multiple z-scores. In this case, we calculated the average of the z-scores of gene expression levels in each pathway.

(2) $$Average\;z-scores=\frac{1}{n}\overset{n}{\underset{i=1}{\sum }}z-score_{i}$$

where *n *is the number of samples of a gene in a pathway.

## Results and discussion

**Figure 3:**
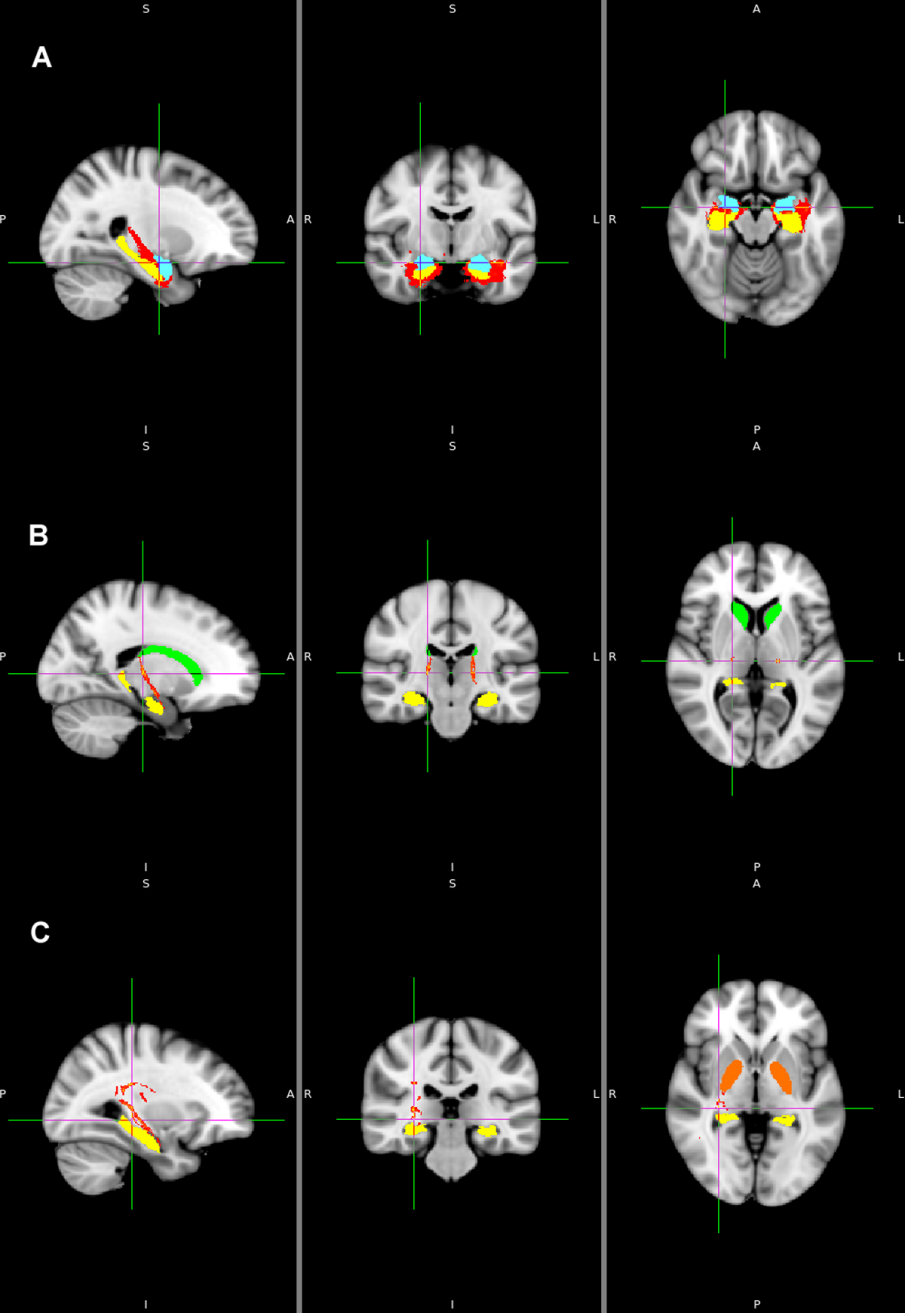
**The fiber pathways from the hippocampus**. 3A: the fiber pathways (red) from the hippocampus to the amygdala (sky-blue), 3B: the fiber pathways (red) from the hippocampus to the caudate nucleus (yellow-green), 3C: the fiber pathways (red) from the hippocampus to the globus pallidus (orange). *Left*: Sagittal view taken in planes parallel to the plane running through the nose keeping the eyes on both sides. *Middle*: coronal view taken in planes parallel to the plane intercepting both ears vertically. *Right*: axial views taken in planes perpendicular to spinal cord axis. P: posterior, A: anterior, R: right, L: left, S: superior, I: inferior.

**Figure 4:**
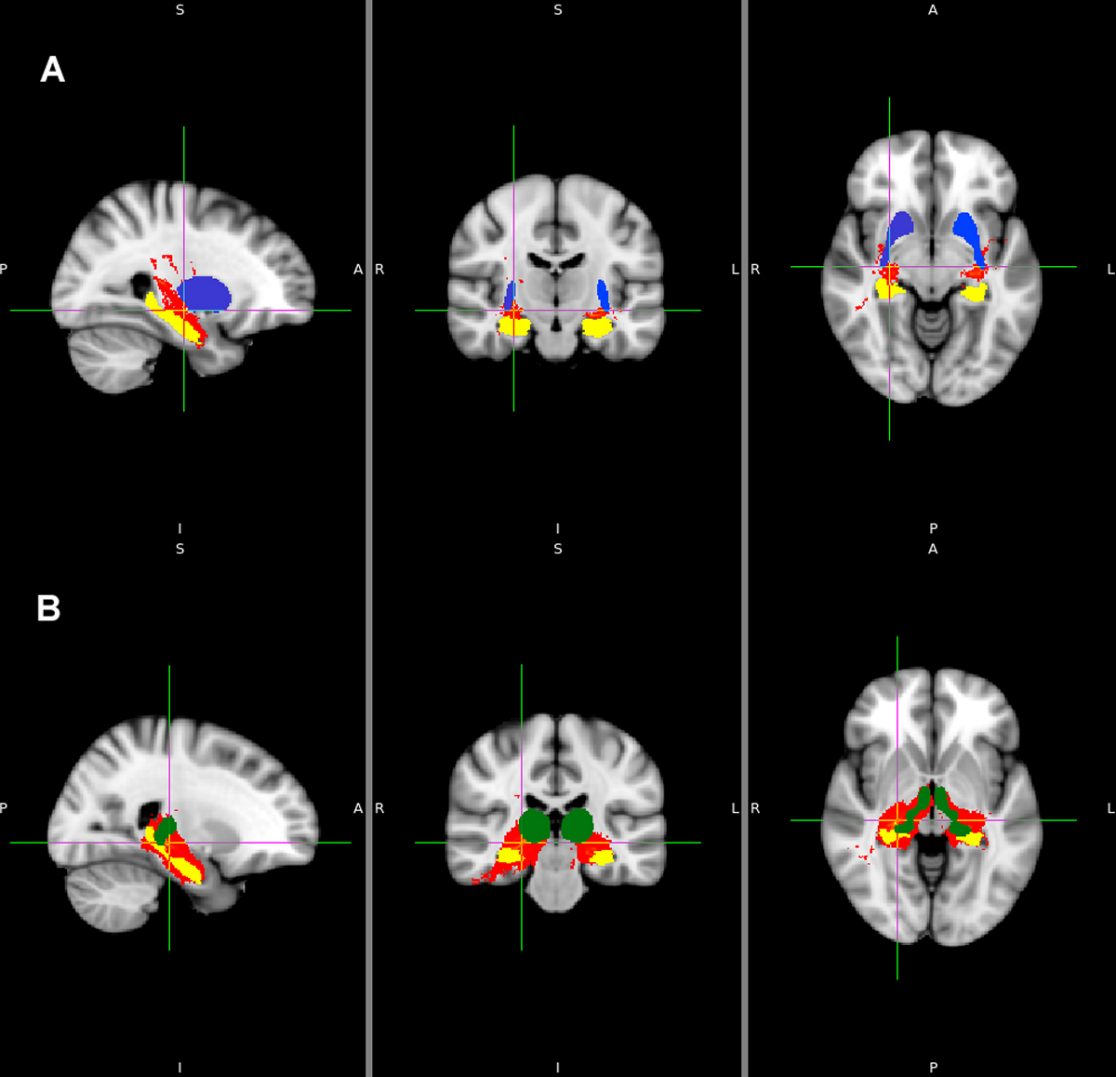
**The fiber pathways the hippocampus**. 4A: the fiber pathways (red) from the hippocampus (yellow) pathway to the putamen (blue), 4B: the fiber pathways (red) from the hippocampus (yellow) pathway to the thalamus (green). *Left*: sagittal view taken in planes parallel to the plane running through the nose keeping the eyes on both sides. *Middle*: coronal view taken in planes parallel to the plane intercepting both ears vertically. *Right*: axial views taken in planes perpendicular to spinal cord axis. P: posterior, A: anterior, R: right, L: left, S: superior, I: inferior.

We traced 12 fiber pathways from the left and right hippocampuses to 6 target regions in each hemisphere. No fiber pathways were found from the hippocampuses to the nucleus accumbens. Table 1 shows 10 fiber pathways of the hippocampuses. Figures 3 and 4 show the pathways visualized by FSL. Figure 3 shows the pathways (red) from the hippocampus (yellow) to the amygdala (sky-blue), the caudate nucleus (yellow-green) and the globus pallidus (orange). Figure 4 shows the pathways (red) from the hippocampus to the putamen (blue) and the thalamus (green). Figure 3A shows the left and right pathways from the hippocampus to the amygdala on the MNI152-T1 1mm template. Likewise, Figures 3B, C, 4A and 4B show the pathways from the left and right hippocampus to target regions.

**Table 1:** 10 fiber pathways of the hippocampus found in the human brain

Seed region	Target region	#voxels	#samples	Figure
Hippocampus, Left (Lh)	Amygdala, Left (Lamy)	13,141	39	3A
Hippocampus, Right (Rh)	Amygdala, Right (Ramy)	9,472	7	3A
Hippocampus, Left (Lh)	Caudate Nucleus, Left (Lcau)	294	0	3B
Hippocampus, Right (Rh)	Caudate Nucleus, Right (Rcau)	572	2	3B
Hippocampus, Left (Lh)	Globus Pallidus, Left (Lpal)	238	1	3C
Hippocampus, Right (Rh)	Globus Pallidus, Right (Rpal)	1,811	1	3C
Hippocampus, Left (Lh)	Putamen, Left (Lput)	2,944	10	4A
Hippocampus, Right (Rh)	Putamen, Right (Rput)	6,405	8	4A
Hippocampus, Left (Lh)	Thalamus, Left (Ltha)	13,884	44	4B
Hippocampus, Right (Rh)	Thalamus, Right (Rtha)	24,309	50	4B

**Figure 5:**
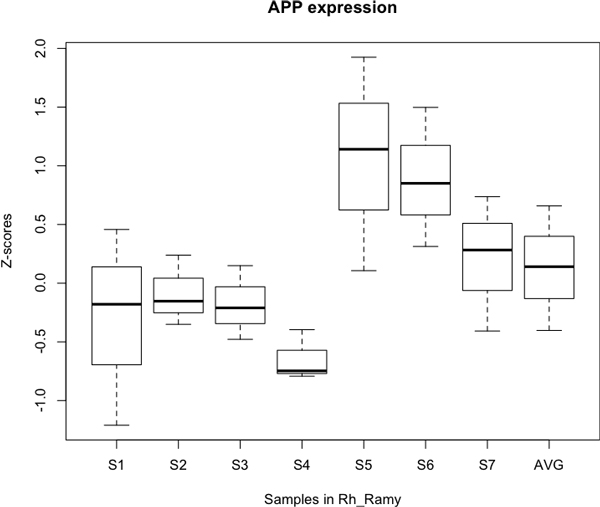
**The z-scores of the APP**. The z-scores of the gene expression levels of the amyloid precursor protein (APP) in the 7 samples located in the pathway from the right hippocampus to the right amygdala. Different samples show different expression levels for APP. AVG represents the average z-score of the 7 samples.

**Figure 6:**
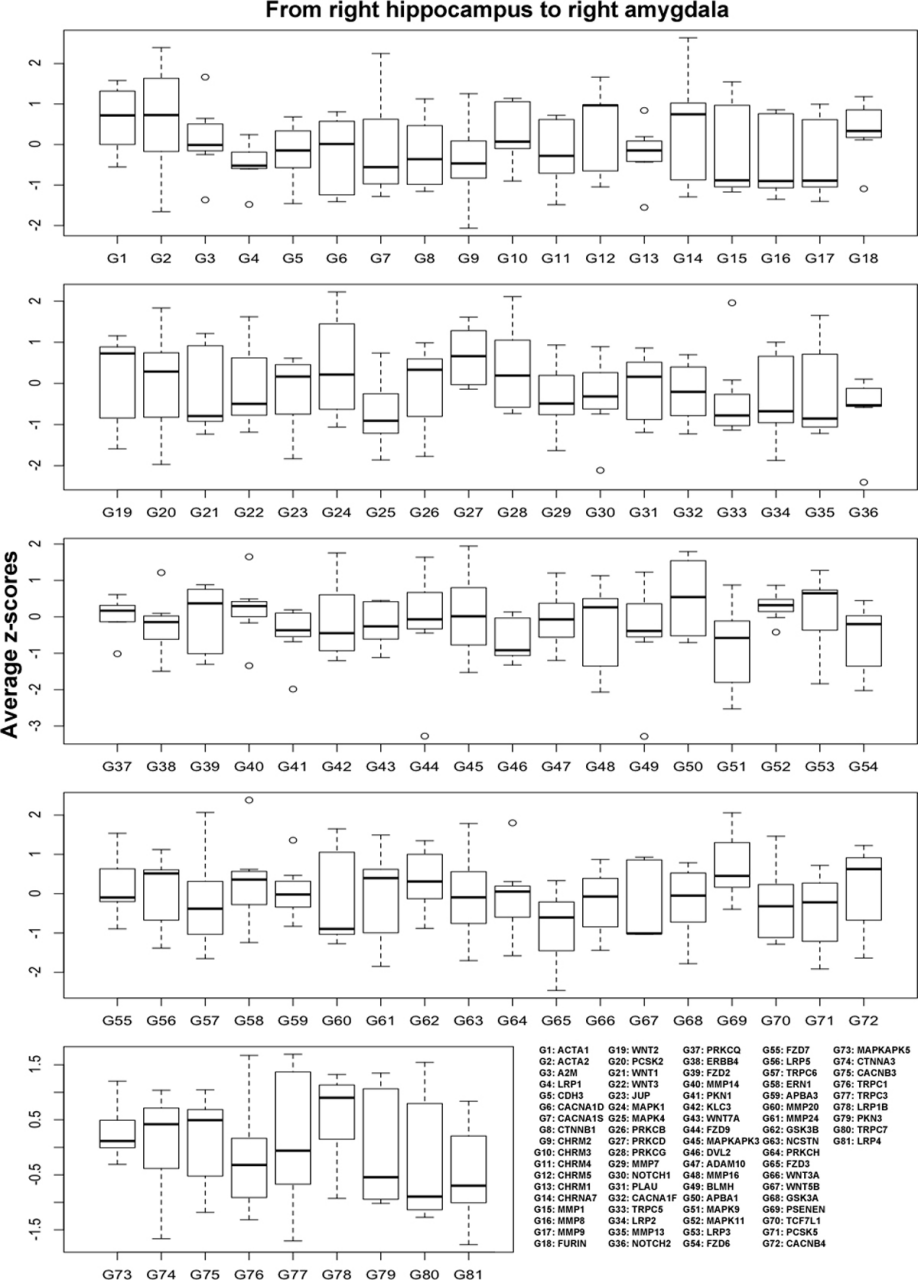
**The average z-scores of Alzheimer-related genes in a fiber pathway**. The pathway from the right hippocampus to the right amygdala has 81 genes. The boxplots show the average z-scores of the expression levels of the 81 genes in a fiber pathway.

Seven samples were found in the pathway from the right hippocampus to the right amygdala. Figure 5 shows the z-scores of the expression of the amyloid precursor protein (APP) in each of the 7 samples. APP is known to induce the amyloid beta [7], which is directly related to Alzheimer's disease. The figure shows different expression levels of the samples. The AVG in Figure 5 represents the average z-scores of the 7 samples. Among the 473 genes that are related to Alzheimer's disease, 81 genes have protein-protein interaction data available at HPRD. Figure 6 shows the average z-scores of the expression levels of the 81 Alzheimer-related genes. G50 in Figure 6, for example, represents the Amyloid beta A4 precursor protein-binding family A member 1 (APBA1), which directly interacts with APP [13,14].

**Figure 7:**
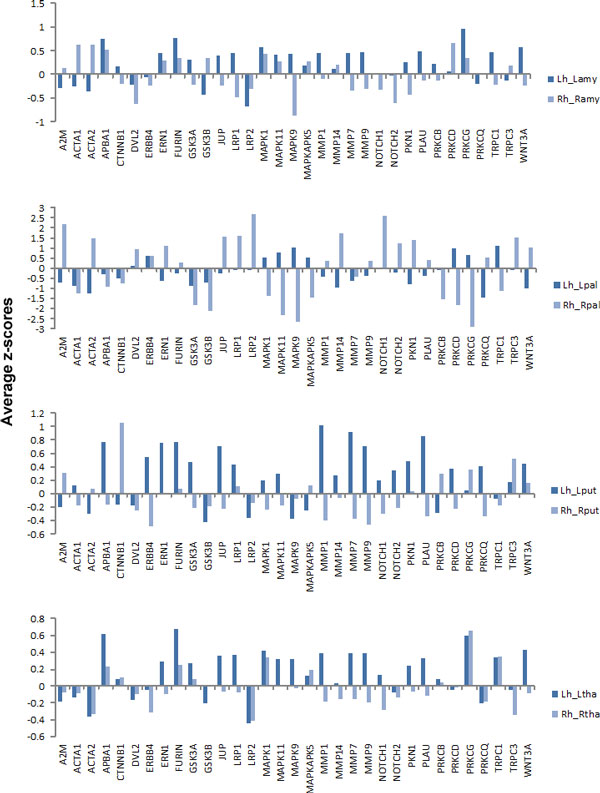
**The average z-scores of the expression levels of the 33 Alzheimer-related genes**. The average z-scores of the expression levels of the 33 Alzheimer-related genes that have more than 10 protein-protein interactions.

**Figure 8:**
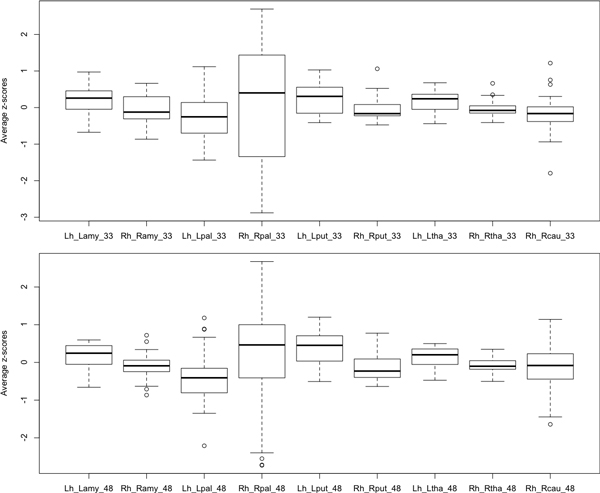
**The average z-scores of the expression levels of all known Alzheimer-related genes**. The 33 Alzheimer-related genes with more than 10 PPIs reveal different expression levels from those of the remaining 48 Alzheimer-related genes. In both groups of genes, expression levels in the left and right pathways are different.

**Figure 9:**
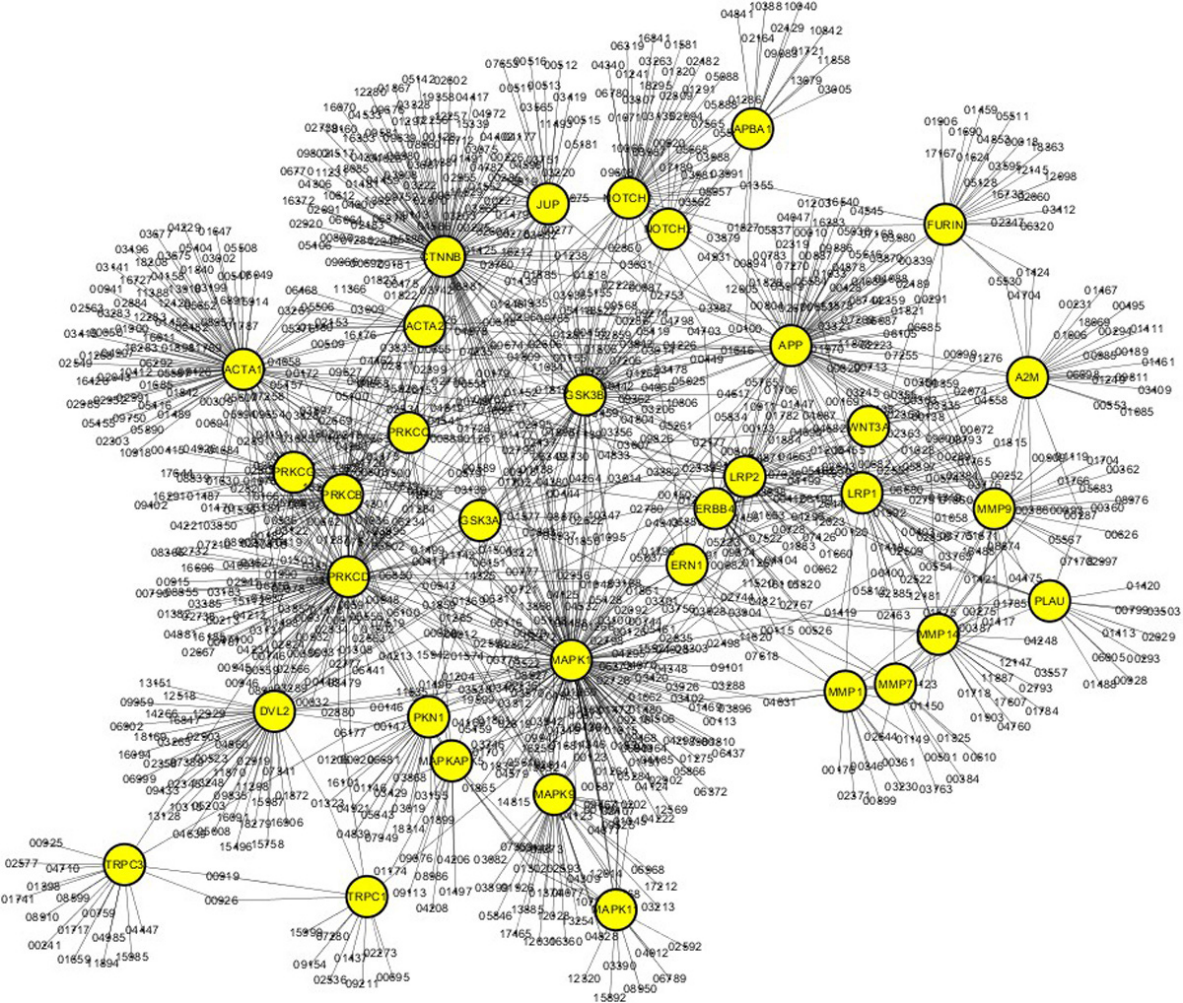
**The protein-protein interaction network of the 33 Alzheimer-related genes**. The network includes 33 Alzheimer-related genes that have more than 10 protein-protein interactions.

Among the 81 Alzheimer-related genes, we selected 33 genes that have more than 10 protein-protein interactions (Table 2). Figure 7 shows average z-scores of the expression levels of the 33 genes in the left and right fiber pathways. As shown in Figure 7, all the genes have different expression levels in the left and right fiber pathways of the hippocampus. The 33 genes also have different expression levels from the remaining 48 genes (Figure 8). Figure 9 shows the protein-protein interaction network of the 33 Alzheimer-related genes (yellow nodes) that have more than 10 protein-protein interactions.

**Table 2:** 33 Alzheimer-related genes with many PPIs

	Gene	HPRD	#PPIs		Gene	HPRD	#PPIs
1	A2M	HPRD_00072	28	18	MAPKAPK5	HPRD_09467	19
2	ACTA1	HPRD_00030	91	19	MMP1	HPRD_00384	16
3	ACTA2	HPRD_00031	15	20	MMP14	HPRD_02856	20
4	APBA1	HPRD_03879	15	21	MMP7	HPRD_01525	16
5	CTNNB1	HPRD_00286	135	22	MMP9	HPRD_00387	31
6	DVL2	HPRD_03690	53	23	NOTCH1	HPRD_01827	46
7	ERBB4	HPRD_02767	29	24	NOTCH2	HPRD_02606	16
8	ERN1	HPRD_04943	12	25	PKN1	HPRD_03019	24
9	FURIN	HPRD_00653	27	26	PLAU	HPRD_01883	16
10	GSK3A	HPRD_06002	21	27	PRKCB	HPRD_01499	68
11	GSK3B	HPRD_05418	74	28	PRKCD	HPRD_01501	103
12	JUP	HPRD_01414	38	29	PRKCG	HPRD_01502	48
13	LRP1	HPRD_00138	55	30	PRKCQ	HPRD_02710	24
14	LRP2	HPRD_02509	44	31	TRPC1	HPRD_11894	13
15	MAPK1	HPRD_01496	161	32	TRPC3	HPRD_15999	19
16	MAPK11	HPRD_04208	15	33	WNT3A	HPRD_05897	12
17	MAPK9	HPRD_04206	39				

**Figure 10:**
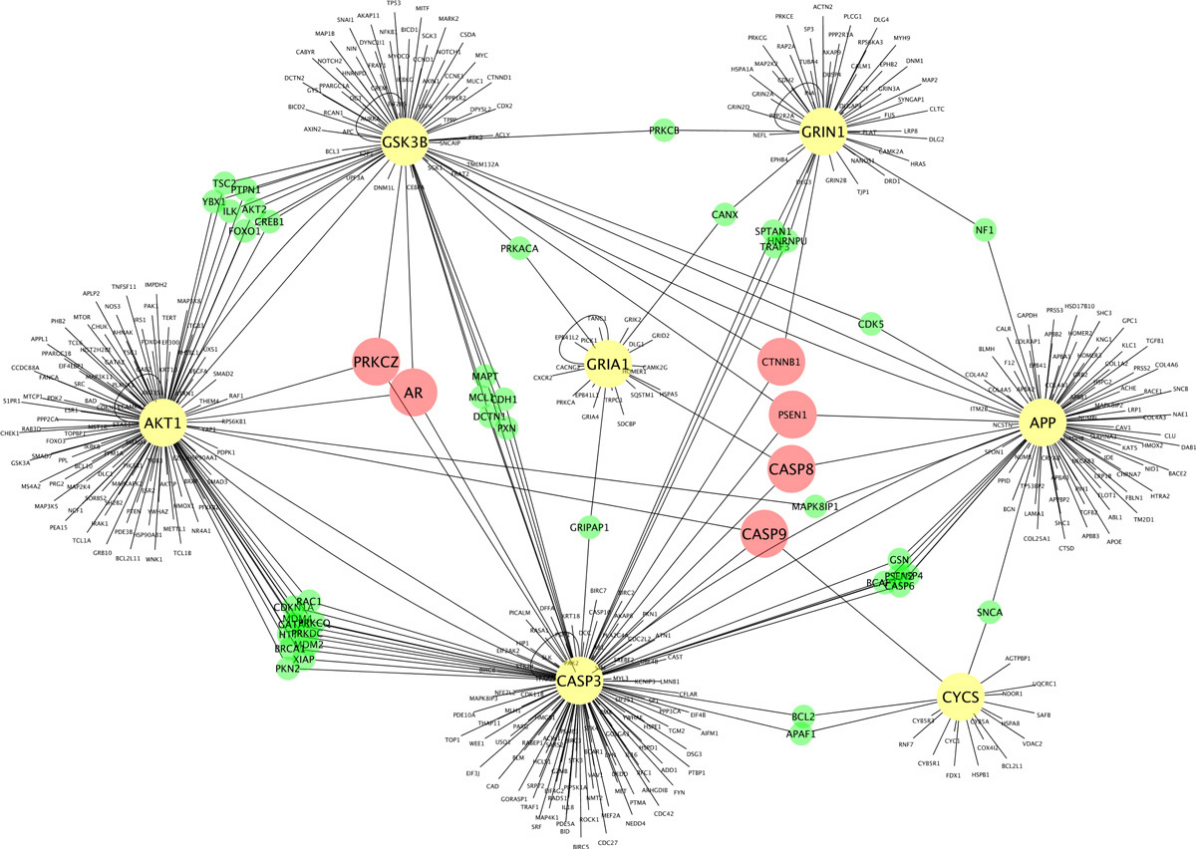
**Modeling the interactions of Alzheimer-related genes from the fiber pathways**. Yellow nodes (AKT1, CASP3, CYCS, GRIA1, GRIN1, and GSK3B) represent the proteins related to the amyloid precursor protein (APP). Red nodes (AR, PRKCZ, CTNNB1, PSEN1, CASP8, and CASP9) represent the proteins that interact with three APP-related proteins (KT1, CASP3, CYCS, GRIA1, GRIN1, and GSK3B). Green nodes represent the proteins that interact with two proteins among APP, AKT1, CASP3, CYCS, GRIA1, GRIN1, and GSK3B. The remaining nodes represent the proteins that interact with the APP-related proteins only. Detailed information of the proteins is available in Additional file 1.

The protein-protein interaction network in Figure 10 includes four types of proteins. Yellow nodes (AKT1, CASP3, CYCS, GRIA1, GRIN1, and GSK3B) represent the proteins related to the amyloid precursor protein (APP). Finding CASP3 and GRIA1 as APP-related proteins agrees with a recent study result that activation of caspase-3 (CASP3) via mitochondria is required for the long-term depression and AMPA (GRIA1 in the network) receptor internalization in hippocampal neurons [15]. Red nodes (AR, PRKCZ, CTNNB1, PSEN1, CASP8, and CASP9) represent the proteins that interact with three APP-related proteins (KT1, CASP3, CYCS, GRIA1, GRIN1, and GSK3B). Green nodes represent the proteins that interact with two proteins among APP, AKT1, CASP3, CYCS, GRIA1, GRIN1, and GSK3B. The remaining nodes represent the proteins that interact with the APP-related proteins only. The microarray data was obtained from a male brain, and the node AR in the network represents the androgen receptor which is critical for the development and maintenance of the male sexual phenotype. The average z-scores of the proteins shown in red (AR, PRKCZ, CTNNB1, PSEN1, CASP8, CASP9) are available in Additional file 1.

## Conclusions

In this study, we tracked inter-region fibers tracts using a new method for detecting crossing fibers in voxels and streamlined the fibers by probabilistic tractography to generate an inter-region physical connectivity map starting from the hippocampus. The primary result of this study is to investigate the connectivity of the left and right hippocampus with other brain regions. We tracked fiber pathways from the left and right hippocampus to 12 other regions of interest (ROI) of brain. The secondary result of this study is to investigate the microarray gene expression of the fiber pathways and to find protein-protein interactions of the genes, which are related to Alzheimer disease.

Although the microarray data DTI data for a single normal control was analyzed in this study, we plan to investigate more cases in the future study when more data is available. Although preliminary, the genes and their interactions found in our study will be useful to design biochemical experiments for further investigation.

## Competing interests

The authors declare that they have no competing interests.

## Authors' contributions

BP analyzed the human brain microarray data and prepared the first draft of the manuscript. WL analyzed DTI data to track the fiber pathway and prepared the manuscript together. KH supervised the work and rewrote the manuscript. All authors read and approved the final manuscript.

## Supplementary Material

Additional file 1 **Average z-scores of the expression levels of the proteins in Figure **10. The average z-scores of the expression levels of the red nodes (AR, PRKCZ, CTNNB1, PSEN1, CASP8, CASP9) and the protein names of the green nodes in Figure 10. Click here for file
